# Controlled Release of Caffeic Acid and Pinocembrin by Use of nPSi-βCD Composites Improves Their Antiangiogenic Activity

**DOI:** 10.3390/pharmaceutics14030484

**Published:** 2022-02-22

**Authors:** Dina Guzmán-Oyarzo, Jacobo Hernández-Montelongo, Carlos Rosas, Pamela Leal, Helga Weber, Marysol Alvear, Luis A. Salazar

**Affiliations:** 1 Center of Molecular Biology and Pharmacogenetics, Department of Basic Sciences, Scientific and Technological Bioresource Nucleus (BIOREN-UFRO), Universidad de La Frontera, Avenida Francisco Salazar 01145, Temuco 4811230, Chile; dina.guzman@uss.cl; 2 Escuela de Tecnología Médica, Facultad de Ciencias de la Salud, Universidad San Sebastián, General Cruz 1577, Concepción 4030000, Chile; 3 Bioproducts and Advanced Materials Research Center (BioMA), Faculty of Engineering, Universidad Católica de Temuco, Avenida Rudecindo Ortega 02950, Temuco 4813302, Chile; jacobo.hernandez@uct.cl; 4 Department of Physical and Mathematical Sciences, Faculty of Engineering, Universidad Católica de Temuco, Temuco 4813302, Chile; 5 Escuela de Medicina, Facultad de Medicina y Ciencia, Universidad San Sebastián, General Lagos 1163, Valdivia 5110693, Chile; carlos.rosas@uss.cl; 6 Center of Excellence in Translational Medicine (CETM) and Scientific and Technological Bioresource Nucleus (BIOREN-UFRO), Universidad de La Frontera, Temuco 4810296, Chile; pamela.leal@ufrontera.cl (P.L.); helga.weber@ufrontera.cl (H.W.); 7 Department of Agricultural Sciences and Natural Resources, Faculty of Agricultural and Forestry Science, Universidad de La Frontera, Temuco 4810296, Chile; 8 Department of Chemical Sciences and Natural Resources, Faculty of Engineering and Sciences, Universidad de La Frontera, Avenida Francisco Salazar 01145, Temuco 4811230, Chile; marysol.alvear@ufrontera.cl

**Keywords:** antiangiogenic activity, caffeic acid, pinocembrin, polyphenols, HUVECs, controlled release, nPSi/βCD microparticle

## Abstract

Although polyphenols have great pharmacological potential, the main disadvantage is that they have low bioavailability at the desired site. Thus, the use of biocompatible systems for drug delivery is a strategy that is currently gaining great interest. The objective of this study is to evaluate the effect of microencapsulation of caffeic acid and pinocembrin on the antioxidant and antiangiogenic activity of both polyphenols, by the use of nPSi-βCD composite microparticles. For this HUVEC, cells were exposed to H_2_O_2_ and to treatments with polyphenols in solution and loaded in the composite microparticle. The polyphenols were incorporated into a microparticle using nanoporous silicon, chitosan and a β-cyclodextrin polymer as the biomaterial. The evaluation of the antiangiogenic effect of the treatments with polyphenols in solution and microencapsulated was carried out through functional tests, and the changes in the expression of target genes associated with the antioxidant pathway and angiogenesis was performed through qPCR. The results obtained show that the caffeic acid and pinocembrin have an antioxidant and antiangiogenic activity, both in solution as microencapsulated. In the caffeic acid, a greater biological effect was observed when it was incorporated into the nPSi-βCD composite microparticle. Our results suggest that the nPSi-βCD composite microparticle could be used as an alternative oral drug administration system.

## 1. Introduction

The generation of new blood vessels is essential and necessary for many physiological processes [1]. Angiogenesis it is defined as the formation of new blood vessels from pre-existing vessels [2] to provide nutrients and oxygen to forming tissues [3]. It is a complex process regulated by the coordinated expression of proangiogenic and antiangiogenic factors [4]. From a pathological perspective, exacerbated vascular growth and inadequate vessel preservation result in ischemic processes that lead to the onset of pathologies, such as cardiovascular diseases [5,6]. Atherosclerosis is a cardiovascular disease that evolves silently and results in the formation of atheromatous plaque. The advance of an atherosclerotic lesion causes many atheromatous plaques to produce areas of calcification [7,8], a process associated with the development of stable or unstable atherosclerotic plaques, which are at the greatest risk of rupture [9]. The main characteristic of an unstable plaque is the presence of intraplaque neo-angiogenesis or neovascularization (NV) [10,11], which occurs as a response mechanism to the progressive increase in the size of the plaque, increase in oxidative stress and inflammation, and the appearance of areas of intraplaque hypoxia. However, the advance of the lesion leads to an imbalance between the antiangiogenic and proangiogenic factors, stimulating the formation of new vessels even further [12], which are characterized as being immature and fragile, and much larger than normal capillaries; they have a thin wall due to the lack of pericytes, they do not have structural support because they have a small number of smooth muscle cells, and they lack complete binding between endothelial cells [13], which causes the new vessels to break easily [14]. On this basis, atheromatous plaques were studied in patients with unstable chest angina who had developed NV, and it was observed that these lesions had all the properties of vulnerable plaques compared to those lesions where there was no NV [15].

The increase in oxidative stress and the presence of radical oxygen species (ROS) in atheromatous plaque promote the immature process of angiogenesis. In this sense, H_2_O_2_ is a key molecule that participates in the activation of a redox signaling mechanism formed by Nrf2/Keap-1 [16]. In conditions of cellular homeostasis, the level of Nrf2 and its activation are controlled mainly by Keap-1 [17]. In a state of oxidative stress, however, Nrf2 is translocated to the nucleus and interacts with transcriptional cis-regulatory elements called antioxidant response elements (AREs), inducing the expression of different target genes involved in cytoprotective processes [18], such as heme oxygenase-1 (HMOX1), GCL, and GSTs. Nrf2 can be induced by many molecules, such as ROS, electrophilic compounds, such as sulforaphane, or tert-butylhydroquinone, and metallic ions [19]. In addition to the activation of the Nrf2/Keap1/ARE system, a control drive has been reported that is independent of the protein repressor Keap1. This mechanism regulates the expression of ARE-dependent genes through the phosphorylation of the proteins Nrf2 and Keap-1 by activating different signal transduction pathways, such as those of mitogen-activated protein kinases (MAPKs), including ERKs (extracellular signal-regulated kinases), JNKs (c-Jun N-terminal kinases), phosphatidylinositol 3-kinase (PI3K/Akt), and protein kinase C (PKC) [20].

Some studies have shown that in cell culture, polyphenols at a very low concentration generate low concentrations of H_2_O_2_, which increases cell proliferation, wound repair, and survival; however, at high concentrations, due to the high H_2_O_2_ production, they inhibit proliferation, angiogenesis, wound repair, and reduce cell survival [21]. Moreover, it was found that preconditioning the cells with H_2_O_2_ modulates the phase II enzymes by activating PI3K/Akt kinase (phosphatidylinositol 3-kinase/protein kinase B), MAPK, and JNK [22,23].

Thus, neo-angiogenesis is a crucial process in the progression of the atherosclerotic lesion, as it contributes to and increases the production of unstable atherosclerotic plaques that are at greater risk of rupture [11,24], which ultimately induces the onset of serious clinical conditions. Therefore, angiogenesis represents an alternative therapeutic target, and, in this context, polyphenol use has gained importance due to its great pharmacological potential. The biological properties reported for polyphenols include relaxants, since they can cause vasodilation in endothelial cells [25]; antioxidants, because they eliminate radicals, such as superoxide and peroxyl radicals, in addition to inhibiting a variety of enzymes responsible for the production of free radicals [26]; antithrombotics, because they inhibit tissue factor activity in peripheral blood monocytes as well as thrombin activity [27]; antiangiogenics, because they inhibit endothelial cell proliferation, migration, and morphogenic differentiation [28]; anti-inflammatories [29]; and anticarcinogenics [30]. Several studies reported that polyphenols, such as quercetin, luteolin, curcumin, caffeic acid, and phenyl ester, exhibit a great antiangiogenic capacity [31], which has been linked mainly to the degree of antioxidant activity that these molecules present. Another study described the neuroprotective and anti-inflammatory effects of resveratrol in rats subjected to brain injury by radiation [32]. Although polyphenols have multiple biological actions, there is reported evidence that polyphenols have low pharmacokinetics, suggesting low bioavailability at the desired site [33]. Therefore, the use of biomaterials is a strategy that was explored to improve the pharmacokinetics and pharmacodynamics of molecules for therapeutic use [34,35,36,37]. Thus, from the available evidence, the general aim of this study is to evaluate, in vitro, the antiangiogenic and antioxidant effects of the polyphenols caffeic acid and pinocembrin in solution and microencapsulated using nPSi-βCD composites. These composite microparticles were recently developed by our group using nanoporous silicon (nPSi), chitosan, and a β-cyclodextrin polymer (βCD polymer) [38]. We studied their use for the controlled release of both caffeic acid and pinocembrin, two main polyphenolic compounds found in Chilean propolis. In that sense, when the flexible and soft βCD polymer is combined within the highly porous inorganic matrix of nPSi microparticles as substrate, the obtained composite extend the interactions with polyphenols, enhancing their stability and controlled release.

## 2. Materials and Methods

### 2.1. Materials

Caffeic acid (CA, MW ≈ 180.16 g/mol), pinocembrin (Pin, MW ≈ 256.25 g/mol), chitosan (Chi, 75–85% deacetylated, low M_W_ ≈ 5 × 10^4^ g/mol), β-cyclodextrin (βCD, M_W_ ≈ 1134.98 g/mol), citric acid (M_W_ ≈ 210.14 g/mol), g NaH_2_PO_2_·H_2_O (M_W_ ≈ 105.99 g/mol), and phosphate-buffered solution (PBS) 0.01 M (0.138 M NaCl, 0.0027 M KCl, pH = 7.4 at 25 °C) were purchased from Sigma-Aldrich, St. Louis, MO, USA. Acetone (C_3_H_6_O), isopropanol (C_3_H_7_OH), ethanol (EtOH, C_2_H_5_OH), glacial acetic acid (CH_3_COOH), hydrogen peroxide (H_2_O_2_), sodium hydroxide (NaOH), hydrochloric acid (HCl), and hydrofluoric acid (HF) were acquired from Merck, Darmstadt, Germany. All chemicals were used without further purification, and solutions were prepared using Milli-Q water with a resistivity of 18.2 MΩcm (pH ~7.6, unless otherwise mentioned). Silicon (Si) wafers (p^+^ type, boron-doped, orientation <100>, resistivity of 0.001–0.005 Ω·cm) were purchased from University Wafer, South Boston, MA, USA. Fetal bovine serum (FBS), L-glutamine, penicillin-streptomycin solution, and D-PBS and Matrigel were purchased from Corning, Manassas, VA, USA. CellTiter-FluorTM assay and the CellTiter 96^®^ AQueous One Solution cell proliferation assay (MTS) was acquired from Promega, Madison, WI, USA. Dimethylsulfoxide 100% (DMSO), CM-H2DCFDA, DNase I and Trizol Ambion were purchased from Thermo Fisher Scientific, Waltham, MA, USA. EvaGreen plus (ROX) qPCR Master Mix was acquired from Biotium, Fremont, CA, USA. iScript Reserve Transcription Supermix kit (RT-PCR), 2-mercaptoetanol were purchased from Biorad, Hercules, CA, USA.

### 2.2. The Synthesis of nPSi-βCD Composites 

nPSi-βCD composite microparticles were synthesized following a method previously reported [38]. Si wafers were cleaned by ultrasonication in acetone, isopropanol, and distilled water. Then, nPSi layers were fabricated by electrochemical etching from the cleaned Si wafers in a HF (48%): ethanol (1:2) solution under a current density of 80 mA/cm^2^ for 30 min. Then, an electropolishing pulse was applied to obtain free-standing nPSi layers. Furthermore, the applied current density was enhanced to 150 mA/cm^2^ during 2 s. nPSi free-standing layers were scraped with a diamond tip to obtain microparticles. They were milled, collected in ethanol, and subjected to 10 min ultrasound agitation for homogenization. Finally, the obtained nPSi particles were chemically oxidized by H_2_O_2_ for 12 h in orbital agitation and rinsed with ethanol. The oxidized nPSi microparticles were immersed in an acid chitosan solution for 15 min and rinsed afterwards with ethanol (nPSi-CHI). The chitosan solution (1% *w*/*v*) was previously prepared with chitosan powder in 100 mM of glacial acetic acid, then the pH value was adjusted at 4 with a 0.1 M HCl and/or NaOH solution. For the composites (nPSi-βCD) synthesis, a monomer solution was prepared with 10 g of βCD, 3 g of NaH_2_PO_2_·H_2_O as catalyst, and 10 g of citric acid in 100 mL of distilled water. Then, nPSi-CHI was immersed in this solution for 15 min while stirring. Samples were dried, first at room temperature, and later at 90 °C for 1 h in each case. The βCD–citric acid in situ polymerization in nPSi-CHI was carried out at 140 °C for 25 min. Subsequently, the samples were rinsed with ethanol, dried at 90 °C for 1 h, and milled for homogenization. Finally, the polyphenols caffeic acid and pinocembrin were loaded into the composite microparticle. Both the synthesis of the composite microparticle and the polyphenol load were evaluated and characterized physically and chemically.

### 2.3. Physicochemical Characterization

The morphology of the microparticles was observed by a variable pressure scanning electron microscope (VP-SEM, SU-3500 Hitachi, Tokyo, Japan) using an acceleration voltage of 5 kV. The zeta potential of microparticles was measured by a ZetaSizer Nano–ZS (Malvern Ltd., Royston, UK), in distilled water.

### 2.4. Loading and Release of Polyphenols

The loading and release of polyphenols using nPSi-βCD composites microparticles were obtained following a method previously reported [38]. Polyphenols CA and Pin were reconstituted with 100% DMSO and stored at −20 °C until required. nPSi-βCD composite microparticles were loaded with CA and Pin using of concentrated aqueous solutions of each polyphenol and placed in a horizontal shaker incubator (NB-2005LN Biotek, Winooski, VT, USA) for 12 h at 50 RPM and room temperature. After polyphenol loading, the samples were rinsed to remove the unentrapped molecules; they were dried at room temperature and milled for homogenization. Polyphenol release in the different biological assays was performed at pH of 7.4.

### 2.5. Cell Culture

For cell culture, human umbilical vein endothelial cells (HUVECs) were obtained from Cell Applications Inc. (San Diego, CA, USA), and maintained in an endothelial cell growth medium (Cell Applications, San Diego, CA, USA) supplemented with 10% FBS, 1% L-glutamine, and 1% penicillin-streptomycin solution. The cell culture was routinely grown under specific conditions in a humidified atmosphere incubator of 95% air and 5% CO_2_ at 37 °C and pH 7.4. Cells were used at no more than seven passages.

### 2.6. Cytotoxicity Assays

For the in vitro viability assays, a CellTiter 96^®^ AQueous One Solution Cell Proliferation Assay (MTS) (Promega, Madison, WI, USA) was used to determine the toxic effect of H_2_O_2_ on HUVEC viability. The MTS assay is based on the conversion of a tetrazolium salt to a colored aqueous soluble formazan product by the mitochondrial activity of viable cells at 37 °C. The amount of formazan produced by dehydrogenase enzymes is directly proportional to the number of living cells in the culture. The viability assays were performed according to the manufacturer’s protocols. Briefly, HUVECs were placed into 96-well plates (2.5 × 10^3^ cells/per well) in 100 μL and incubated at 37 °C. Then, the cells were exposed to increasing concentrations of H_2_O_2_ (0.2–50 mM). After 12 h of incubation, the medium was removed and 20 μL of MTS reagent was added to the wells, followed by incubation for 4 h at 37 °C and pH 7.4. The absorbance was determined by a microplate reader (NanoQuant, Infinite^®^ M200PRO—Tecan) at 490 nm. The results were expressed as the percentage of viability relative to the control. The cell viability was calculated as follows: cell viability (%) = (OD of treatment group/OD of control group) × 100.

### 2.7. Oxidative Stress Induction

The CM-H2DCFDA probe was used to evaluate the amount of oxidative stress generated by the treatment with different concentrations of H_2_O_2_. Briefly, 2 × 10^5^ cells/well were seeded in a final volume of 500 μL for 24-well cell plates. The plate was maintained in culture for a period of 18–24 h, then 300 μL of PBS was added to each well with the CM-H_2_DCFDA and it was left to incubate in darkness for 40 min at 37 °C and pH 7.4. Next, the PBS was discarded and the cells were treated with different concentrations of H_2_O_2_. Once the treatment time had passed, the medium with treatment was eliminated and it was washed once with PBS. A total of 250 μL of trypsin was added to each well to remove the cells. Trypsin was inactivated with 500 μL of FBS to then collect the cells in tubes for cytometry. The tubes were centrifuged at 800 rpm for 8 min, the supernatant was discarded by inverting the tube, and 300 μL of cold PBS was added to wash the cells. Centrifugation was performed again and finally the pellet was re-suspended with 150 μL of cold PBS. The tubes were placed on the cytometer to be read at 492–95 nm/517–57 nm of excitation and emission, respectively.

### 2.8. Evaluation of Antiangiogenic Activity of Caffeic Acid and Pinocembrin

#### 2.8.1. Chick-CAM Assay

For the chick chorioallantoic membrane (CAM) assay, the eggs were purchased at the Chilean Institute of Public Health and fertilized as described by Sinning et al. (2012) [39]. Briefly, the fertilized eggs of White Leghorn hens (*Gallus gallus*) were kept in an incubator at 38.2 °C in a humidified atmosphere. The pole (apex) of the embryo was marked and incubated at 37 °C for another 72 h under stable pH conditions of 7.4. Then, a small hole (1 mm) was made in the egg shell at the previously marked pole. Next, at stage 20 of embryonic development, a large opening (2 × 1 cm) was created in the embryonic pole, which was sealed with plastic wrap (Saran Wrap^®^, Racine, WI, USA). One week later, the plastic cover was removed, and a sterile methylcellulose filter 5 mm in diameter soaked with 10 μL of each treatment was placed on the growing chorioallantoic membrane and in direct contact with the vascularized region. After 3 days, the CAMs were removed from the eggs, fixed in 4% p-formaldehyde, then dehydrated, embedded in paraffin, and stained with hematoxylin-eosin. The blood vessels in the histological samples of each group were counted using an optical microscope (Motic BA310) at 400× magnification. The count was performed in 25 microscopic fields by two blinded examiners. The results were expressed as the number of blood vessels per field.

#### 2.8.2. Formation of Tubular Structures in the Matrigel

The ability of HUVECs to form structures similar to blood vessels was assessed by an assay using high growth factor Matrigel (Corning, New York, NY, USA) in the presence of H_2_O_2_ at a previously standardized concentration, and different concentrations of AC and Pin polyphenols both in solution and microencapsulated. This assay was conducted according to a protocol previously described by Arnaoutova et al. (2010) [40] with some modifications. Briefly, one day before beginning the assay, the cells were sub-cultured in the normal way and then 5 × 10^5^ cells were seeded in a T75 flask. A total of 50 µL of Matrigel was loaded per well, then the 96-well plate was left at 37 °C for 30 min so that the Matrigel could gellify. Then, the cells were re-suspended carefully, pipetting 100 µL of the suspension (15,000 cells) per well. The plate was incubated for 4 h with H_2_O_2_ at a concentration of 0.39 mM prior to the treatment with the polyphenols, a treatment which was kept for a period of 8 h under stable pH conditions of 7.4. Photos were taken with an inverted microscope (Olympus). The images were processed with the Image Processing and Analysis in Java software (ImageJ 1.52a, macros Angiogenesis Analyzer (Bethesda, MD, USA).

### 2.9. Determination of the the Antioxidant Activity of Polyphenols

The antioxidant capacity of polyphenols CA and Pin, both in solution and microencapsulated, was determined by means of the ABTS radical method. The decolorization reaction of the radical ABTS was performed using the following pipette scheme: 50 µL of polyphenol + 1450 µL of ABTS radical reagent. The reaction was left to incubate in darkness for 30 min. One minute was timed between each reaction to not vary the reaction time with the radical between each measurement. The absorbance reading was performed at 734 nm using a spectrophotometer. Acetate buffer was used as the target and the absorbance of the radical ABTS was recorded (0.700 ± 0.010). The measurements were taken according to the methodology proposed by Re et al. (1999) [41] with some modifications.

### 2.10. Evaluation of Gene Expression

#### 2.10.1. RNA Extraction

Total RNA extraction was performed from treated HUVECs. For this, 1.5 × 10^5^ cells/plate were seeded with p-60 for each treatment. The cells were stimulated for 4 h with 0.4 mM of H_2_O_2_ and then treated for the following 24 h with different concentrations of polyphenols in solution and microencapsulated under stable pH conditions of 7.4. Total RNA was isolated with Trizol Ambion following the manufacturer’s instructions. RNA samples were spectrophotometrically quantified, characterized by electrophoresis in 1% agarose gel, and then used as templates to generate complementary DNA (cDNA) under standard conditions in the presence of DNase to eliminate any traces of genomic DNA. The synthesis of cDNA was carried out by reverse transcription reaction (RT-PCR) using the iScript Reserve Transcription Supermix kit.

#### 2.10.2. Real Time PCR

An analysis was performed of the expression of genes related to the antioxidant response and angiogenesis (Nrf2, Keap1, Akt, Cat, Glut-P, Hmox1, Gclc, and Gclm) in cells subjected to oxidative stress (H_2_O_2_), and then treated with different concentrations of two polyphenols CA and Pin, in solution and microencapsulated, under stable pH conditions of 7.4, by real-time PCR (qPCR) using the Step One system (Applied Biosystems, Waltham, MA, USA). The qPCR reaction for all the samples was prepared with 4 µL of EvaGreen plus (ROX) qPCR Master Mix (2X, Biotium, Fremont, CA, USA), 1 µL of each primer (forward and reverse) in a concentration of 200 nM, 1 µL of cDNA in a 1:10 dilution, and nuclease-free water until reaching a final volume of 20 μL. The amplification protocol by qPCR consisted of an initial activation at 95 °C for 5 min, followed by 40 cycles that included denaturation at 95 °C for 15 s, hybridization at 60 °C for 20 s, and extension at 72 °C for 20 s. The relative quantification of the gene expression was analyzed using the method for obtaining the threshold cycle (Ct) and then a Ct comparison between the number of transcripts of the gene in the samples and the normalizer gene through the 2^ΔΔ^Ct method.

### 2.11. Statistical Analysis

The results were expressed as the mean and standard deviation and analyzed using GraphPad Prism v9.0.0 (San Diego, CA, USA). Outliers were detected by the ROUT or Grubbs’ test. The data distribution was evaluated using the Anderson–Darling test for normality, the Shapiro–Wilk, D’Agostino and Pearson, or Kolmogorov–Smirnov normality test. The comparison between groups was performed through the *t*-test or ANOVA with a Tukey’s post-test. If it was not possible to fulfill the statistical assumption of normality and homogeneity of variance, the Mann–Whitney or Kruskal–Wallis nonparametric tests were performed with Dunn’s post-test. For the analyses, *p* < 0.05 and *n* = 3 were considered statistically significant.

## 3. Results and Discussion

### 3.1. Hydrogen Peroxide Cytotoxicity

**Figure 1:**
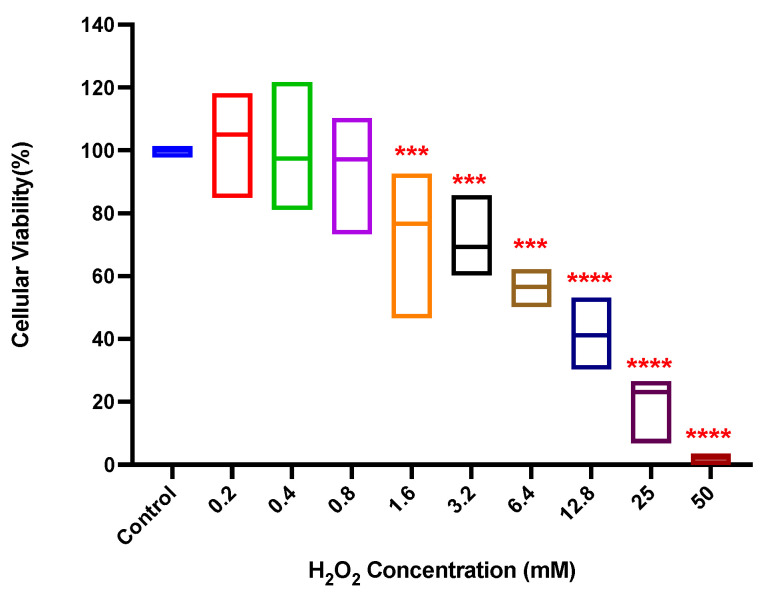
Cell viability of HUVECs treated with H_2_O_2_. The cells were exposed to different concentrations of H_2_O_2_ for 12 h and then the cell viability was measured by MTS assay. Data are presented as the mean ± standard deviation of the results obtained in independent experiments. Statistically significant differences are indicated compared to controls (*** = *p* < 0.001; **** = *p* < 0.0001; *n* = 3).

To evaluate the effect of H_2_O_2_ on HUVEC viability, cells were cultured that were treated with concentrations of H_2_O_2_ between 0.2 and 50 mM for 12 h. The results revealed that cell viability at concentrations of 0.2–0.8 mM varied between 120–78%; however, this variation did not affect the cell (*p* > 0.05). Yet, from 1.6 to 50 mM, a gradual and significant reduction in viability below 80% can be observed (*p* < 0.001) (Figure 1). From these results, it was decided that a concentration of 0.4 mM of H_2_O_2_ would be used to induce oxidative stress in HUVECs, as it did not affect cell viability.

### 3.2. Oxidative Stress Induction

**Figure 2:**
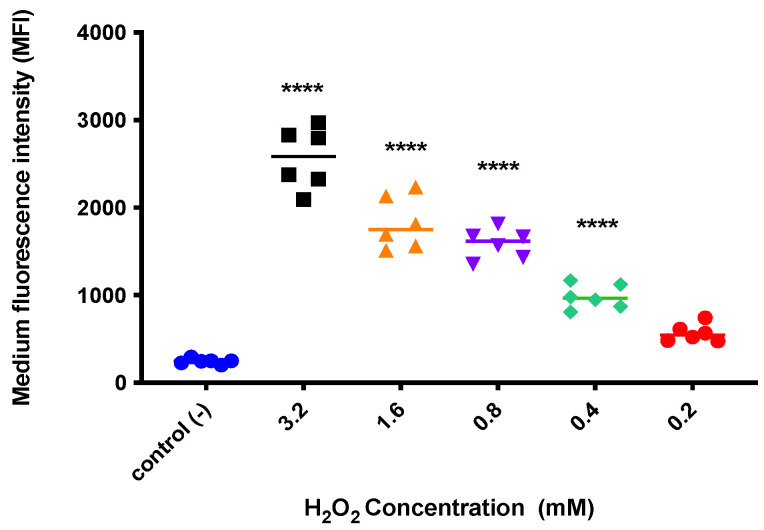
Evaluation of oxidative stress in HUVECs treated with H_2_O_2_. The cells were exposed to different concentrations of H_2_O_2_ for 4 h and the amount of fluorescence emitted was then measured using the CM-H_2_DCFDA probe assay. Data are presented as the mean ± standard deviation of the results obtained in independent experiments. Statistically significant differences are indicated compared to controls (**** = *p* < 0.0001; *n* = 3).

To verify if the selected concentration of H_2_O_2_ induces oxidative stress, the CM-H_2_DCFDA probe was used, for which cells were cultured that were treated with H_2_O_2_ concentrations between 0.2 and 3.2 mM for 4 h. H_2_O_2_ concentrations between 3.2 and 0.4 mM are found to induce oxidative stress significantly in a dose-dependent manner (*p* < 0.0001), i.e., the higher the concentration, the greater the induction of oxidative stress, whereas at 0.2 mM of H_2_O_2_, no differences are noted compared to the control (*p* > 0.05) (Figure 2). In terms of establishing the minimum dose needed to induce oxidative stress, the study results indicate that an optimal concentration would be 0.4 mM of H_2_O_2_, which is supported in the differences to the control in the induction of oxidative stress. Similarly, a study determined that concentrations of up to 0.1 mM H_2_O_2_ maintain viability above 80% of the primary culture osteoblastic cells of rats treated for 24 h [42]. These concentrations are greater than those reported in other studies that determine H_2_O_2_ concentrations to induce the desired effect. In this sense, Bhatia et al. (2017) [43] established that after the treatment with ~60 μM H_2_O_2_, the viability of neuroblastoma cells (IMR32) remained above 80%. The discrepancies observed between the studies could be due to factors inherent in the assays, such as the type of cells used (cell line or primary culture), cell culture conditions specifically with respect to the time at which the cells were exposed to the treatment with H_2_O_2_, or the type of assay used to determine cell viability.

Despite the differences encountered in the viable doses for H_2_O_2_, it is important to emphasize that the dose selected (0.4 mM) in this study was supported in the results from two different cytotoxicity methods and the evaluation of oxidative stress through the CM-H_2_DCFDA probe. On the other hand, the strategy to evaluate the induction of oxida-tive stress could also be contributing to the differences found, because this measures the general production of ROS; therefore, to improve this aspect, it would be highly beneficial to incorporate the specific measurement of the by-products formed from the decomposi-tion of the H_2_O_2_ or the antioxidant enzyme activity (catalase or glutathione peroxidase) in the supernatant of the cell culture. In summary, the differences encountered could be due only to the standardized conditions and the cell model used; therefore, 0.4 mM H_2_O_2_ is the optimal concentration to be able to study it.

### 3.3. The Antiangiogenic Effect of the Treatment with Caffeic Acid and Pinocembrin in Solution on the Formation of Vessels in the Chorioallantoic Membrane of Embryonated Chicken Eggs

**Figure 3:**
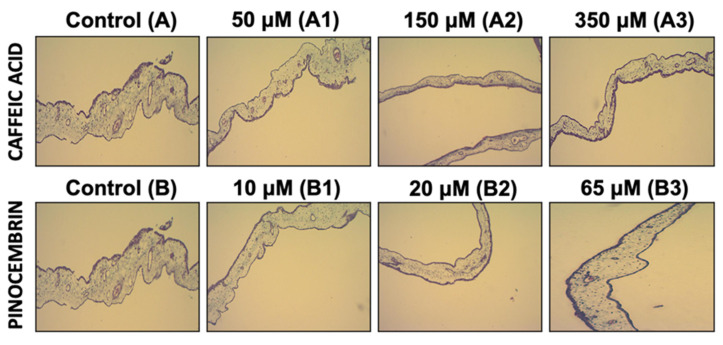
Antiangiogenic effect of treatment with caffeic acid and pinocembrin in solution on the formation of blood vessels in the chorioallantoic membrane of embryonated chicken eggs. (**A1**–**A3**): Representative images of formation of blood vessels in embryonated chicken eggs treated with increasing concentrations of caffeic acid. (**B1**–**B3**): Representative images of formation of blood vessels in embryonated chicken eggs treated with increasing concentrations of pinocembrin.

**Figure 4:**
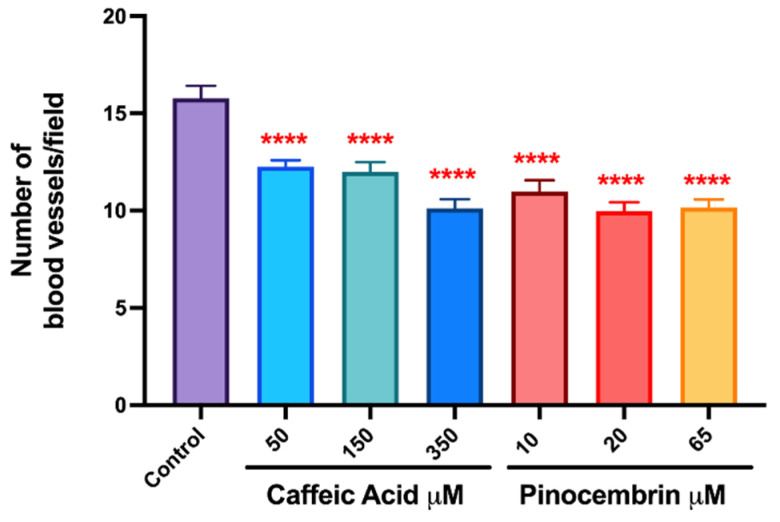
Antiangiogenic effect of treatment with caffeic acid and pinocembrin on the formation of vessels in the chorioallantoic membrane of embryonated chicken eggs. Three sections per egg were analyzed, blood vessels were photographed and counted per mm^2^ of optical field. The numerical data show the mean ± standard deviation of the results obtained in independent experiments. Statistically significant differences are indicated compared to controls (**** = *p* < 0.0001; *n* = 3).

To evaluate the antiangiogenic activity of the polyphenols CA and Pin, an in vivo model was made of angiogenesis in the chick chorioallantoic membrane (CAM). For this, embryonated eggs exposed to different concentrations of CA and Pin, of 50, 150, 350 μM and 10, 20, 65 μM, were used, respectively. When the microvascular density was compared in the different experimental groups (control vs. treated), a significant decrease was observed in the number of blood vessels in the group treated with CA and Pin (*p* < 0.0001). For the group treated with CA, the count is 11 ± 2.6 blood vessels/field (Figure 3A1–A3) and for the group treated with Pin, 10 ± 2.9 blood vessels/field (Figure 3B1–B3), whereas for the control group it is 16 ± 3.8 blood vessels/field (Figure 3A,B). Conversely, when comparing the different concentrations tested in the same experimental group, CA or Pin, no differences is observed. No significant difference is found between the group treated with CA vs. Pin either (*p* > 0.05) (Figure 4). Although it was demonstrated that CA and Pin present antiangiogenic activity, this assay specifically evaluated the antiangiogenic activity of these polyphenols in solution; however, it would be interesting to continue this study by evaluating the antiangiogenic effect of these treatments on polyphenols loaded in the nPSi-βCD composite microparticle, since the evidence indicates the utility of the CAM assay in the study and evaluations of the in vivo properties of biomaterials before moving on to the animal model [44,45]. In this context, the antiangiogenic activity of estradiol inside polyurethane (PU) was evaluated, and the CAM assay revealed a significantly greater angiogenic potential when 17-β-estradiol was incorporated within the biomaterial for its use in the repair of the pelvic floor [46]. In addition, several biocompatible materials and their angiogenic properties were tested, achieving very good results, and illustrating the great applicability of this assay [47].

### 3.4. The Synthesis of Composite Microparticles nPSi-βCD and Physicochemical Characterization

**Figure 5:**
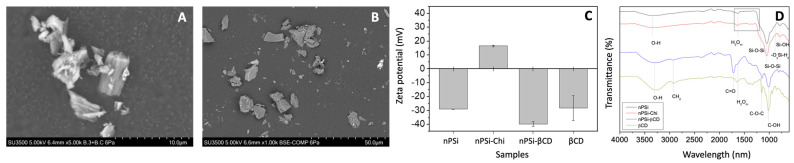
Monitoring of the synthesis process of nPSi-βCD composite microparticles: (**A**) Scanning electron microscope images (SEM) of samples nPSi, (**B**) Scanning electron microscope images (SEM) of samples nPSi-βCD, (**C**) Zeta potential and (**D**) the magnification of ATR-FTIR spectra.

As the synthesis of composite microparticles was obtained by the electrostatic attraction of oppositely charges [38]. SEM images were produced (Figure 5) to analyze the size and morphology of samples at the different stages of synthesis. nPSi and nPSi-Chi presented irregular shapes with an average size of 2.0–2.5 μm (Figure 5A), and both kinds of microparticles showed a rougher surface due to their columnar pores of a ~50 nm width. In the case of the nPSi-βCD sample, the microparticle shapes were also irregular with a larger size of around 14.0 μm, and their faces exhibited a softer appearance (Figure 5B). The increase in the particle size may have occurred because the small particles agglomerated during the polymerization forming higher particles. A similar size distribution of this kind of particle for the oral drug delivery system was previously reported.

A zeta potential analysis was performed (Figure 5C). In the case of nPSi, its negative zeta potential value (−29.06 ± 0.06 mV) would correspond to the negatively charged silanol groups produced by the chemical oxidation with H_2_O_2_. nPSi-Chi showed positive values (16.5 ± 0.6 mV) because the grafting with chitosan would generate a rich aminated surface. On the other hand, the sharp negative zeta potential of nPSi-βCD (−39.8 ± 1.73 mV) was according to the βCD value (−28.2 ± 9 mV), which was generated by the hydrophilic outer surface cavity (C–OH groups) of βCD molecules [48]. 

In that sense, ATR-FTIR analysis was performed to determine the chemical changes of nPSi microparticles during the cascade synthesis processes (Figure 5D). The spectrum of nPSi showed a sharp transmittance peak at 1050 cm^−1^ with a shoulder at 1170 cm^−1^, which both correspond to Si–O–Si stretching mode. Additionally, weak bands at 880 and 795 cm^−1^ related to −OySi-Hx and SiOH, respectively, and the O–H stretching band from SiOH and adsorbed H_2_O at 3350 cm^−1^ were detected. Moreover, the molecular water (H_2_Om) absorbance band was observed at 1630 cm^−1^. These detected functional groups are in agreement with the chemical oxidation of nPSi via H_2_O_2_. On the other hand, the spectrum of nPSi-Chi present the same functional groups as nPSi, plus the weak bands of N–H and amide III detected at 1408 and 1320–1346 cm^−1^. Those bands are related to the polyamino-saccharide chains of Chi, which were used to link the βCD polymer with nPSi microparticles. Regarding the spectrum of nPSi-βCD, the bands corresponding to the spectrum of native βCD were observed: C–OH stretching (1021 cm^−1^), C–O–C stretching (1150 cm^−1^), H_2_Om (1630 cm^−1^), CH_2_ asymmetric stretching (2930 cm^−1^), and O-H stretching from hydroxyl groups (3300 cm^−1^). nPSi-βCD barely showed an extra band in comparison to βCD at 1721 cm^−1^, which corresponded to the C=O groups generated during the polymerization achieved between βCD and citric acid.

In relation to this, nanoporous silicon (nPSi) is an excellent biomaterial that was successfully used for the controlled release of different drugs and biomolecules and, combined with a flexible and soft polymer (β-ciclodextrin) to formed composites, can improve stability and the control of drug release. In recent times, there has been an exponential growth in evidence that supports the usefulness of drug delivery systems, mainly with the aim of improving problems associated with low gastrointestinal absorption and/or instability of phytomolecules, such as polyphenols. In this sense, different phenolic compounds were incorporated into biomaterials, such as polyphenols from green tea extract [49], chokeberry polyphenols [50], and pomegranate peel extract [51]. Consequently, the principle application of our results is that it would allow for the use of this controlled drug release system for future antiangiogenic and antioxidant therapies for chronic non-communicable diseases, such as cancer [52], diabetes [53], and also the cardiovascular diseases, becoming hopeful pharmacological options.

### 3.5. The Effect of the Treatment with Caffeic Acid and Pinocembrin in Solution and Loaded in the nPSi-βCD Composite Microparticle on the Capacity of HUVECs to Form Tubular Structures in Matrigel

**Figure 6:**
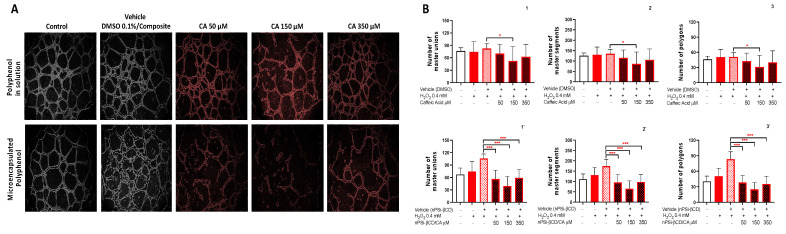
Effect of treatment with caffeic acid in solution and loaded in the nPSi-βCD composite microparticle on the ability of HUVECs to form tubular structures in Matrigel. (**A**) The images were taken at 8 h and are representative for each treatment group. (**B**) Effect quantification of treatment with caffeic acid in solution and loaded in the nPSi-βCD composite microparticle on the ability of HUVECs to form tubular structures in Matrigel. (**1**,**1`**) number of master unions, (**2**,**2`**) number of master segments, and (**3**,**3`**) number of polygons formed. The numbers 1, 2, and 3 correspond to the polyphenol in solution, while 1`, 2`, and 3` correspond to the microencapsulated polyphenol. Statistically significant differences are indicated compared to the control treatment (vehicle). (* = *p* < 0.05; *** = *p* < 0.001; *n* = 3).

To evaluate the antiangiogenic activity, in vitro, of the polyphenols CA and Pin, the test of tubular structure formation was performed in Matrigel (Figure 6). For this, HUVECs + 0.4 mM of H_2_O_2_ were seeded in Matrigel. After 4 h, the different treatments with polyphenols were added both in solution and microencapsulated. To quantify the formation of tubular structures in the matrix, the number of free cell surface extensions, cell surface extensions in contact with other cells, and the closed polygon that formed between the extensions were obtained. Figure 6 shows that the treatment with CA 150 μM in solution significantly reduces the number of unions (control: 80 vs. CA150: 50), segments (control: 130 vs. CA150: 85) and formed polygons (control: 50 vs. CA150: 30), (*p* < 0.05) (Figure 6B1–3). For the other concentrations of CA, 50 and 350 μM, no significant changes were noted compared to the control (vehicle).

These results agree with the report by Paeng et al. (2015) [28], who evaluated the antiangiogenic activity of caffeic acid phenethyl ester (CAPE). Among their results, they highlighted that CAPE strongly inhibited the formation of tubular structures of epithelial cells of human retinal pigment under conditions of hypoxia. Similarly, another study conducted using a HUVEC model demonstrated that ferulic acid (phenolic acid) inhibits cell proliferation, migration, and the formation of tubular structures in response to fibroblast growth factor 1, which proves the antiangiogenic activity of this polyphenol [54].

Alternatively, when the antiangiogenic activity was evaluated, but this time with the polyphenol (CA) loaded in the nPSi-βCD composite microparticle, there was a significant reduction in the formation of tubules in the three concentrations tested (50, 150 and 350 μM), unlike what was found in the polyphenol in solution (Figure 6). However, a greater reduction can be observed in nPSi-βCD + 150 μM CA (Figure 6B1`–3`), compared to the other concentrations of nPSi-βCD + CA 50 and 350 μM CA. Finally, when the treatments between CA in solution and microencapsulated were compared, the results showed a greater antiangiogenic activity of the polyphenol when it was loaded in the microparticle (*p* < 0.001), i.e., the CA encapsulation improved the biological effect of this polyphenol.

**Figure 7:**
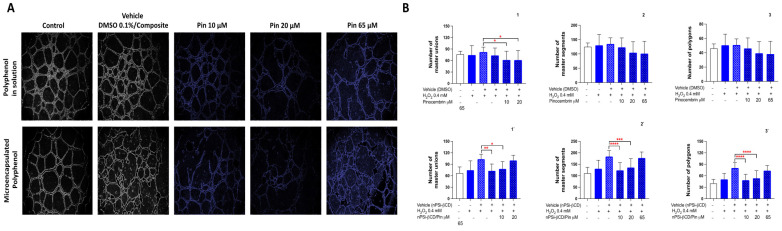
Effect of treatment with pinocembrin in solution and loaded in the nPSi-βCD composite microparticle on the ability of HUVECs to form tubular structures in Matrigel. (**A**) Images were taken at 8 h and are representative for each treatment group. (**B**) Effect quantification of treatment with pinocembrin in solution and loaded in the nPSi-βCD composite microparticle on the ability of HUVECs to form tubular structures in Matrigel. (**1**,**1`**) number of master unions, (**2**,**2`**) number of master segments, and (**3**,**3`**) number of polygons formed. The numbers 1, 2, and 3 correspond to the polyphenol in solution, while 1`, 2`, and 3` correspond to the microencapsulated polyphenol. Statistically significant differences are indicated compared to the control treatment (vehicle). (* = *p* < 0.05, ** = *p* < 0.01, *** = *p* < 0.001, **** = *p* < 0.0001, *n* = 3).

For the case of Pin in solution (Figure 7), the results indicate a significant reduction in the number of master unions in the groups treated with Pin 20 and 65 μM (control: 83 vs. Pin 20 and 65:61, *p* < 0.05) (Figure 7A), whereas in the number of segments and polygons formed, no differences are noted compared to the control in any of the concentrations (*p* > 0.05) (Figure 7B2,3). This is consistent with other studies where the antiangiogenic capacity of other polyphenols, for example analogs of quercetin and luteolin, were studied. The results revealed that quercetin, lutein, and their analogs significantly inhibit cell migration, a key process in angiogenesis [55], and these are in the same family of flavonoids as pinocembrin. Although the evidence described accounts for the antiangiogenic effect of other polyphenols, this study was based on the use of polyphenols that present high concentrations in Chilean propolis from the Region of La Araucanía. In this sense, previous evidence that characterized propolis in the region demonstrated that polyphenols, such as pinocembrin and caffeic acid, are abundant in the propolis of the area [56]. Later, based on an in vitro model (HUVECs) and in vivo model (mouse with atherosclerosis), it was shown that the polyphenols present, including pinocembrin, have anti-atherogenic and antiangiogenic properties [57]. Interestingly, the results of this study, which report on the antiangiogenic effect of pinocembrin, are consistent with the previous evidence described in an in vivo model in terms of the observed biological effect.

When evaluating the ability of the Pin loaded in the nPSi-βCD composite microparticle to form tubular structures, a significant reduction can be observed in the polygonal structures, segments, and master unions formed in the concentrations of nPSi-βCD + Pin 10 and 20 μM (*p* < 0.05) (Figure 7A). Figure 7B1`–3` illustrates that the group treated with nPSi-βCD + Pin 65 μM shows no differences from the control (vehicle). Therefore, a comparison of the groups treated with Pin in solution and microencapsulated provides evidence of an improvement in the antiangiogenic activity, mainly at the lowest concentrations used. In this regard, some authors examined the correlation between the antioxidant and antiangiogenic activity of a polyphenol group of propolis, finding that the greater the antioxidant capacity of the polyphenol, the greater the antiangiogenic activity [58]. In that sense, the polyphenols that belong to the flavonoid group (pinocembrin) have a lower antioxidant activity than phenolic acids (caffeic acid), which would partly explain the differences found between CA and Pin in solution and loaded nPSi-βCD microparticle. Finally, the results demonstrate that the use of controlled drug-release systems, as in the case of the nPSi-βCD composite microparticle, improve the antiangiogenic activity of pinocembrin and caffeic acid.

### 3.6. Antioxidant Capacity of Polyphenols in Solution

The capacity of CA and Pin to remove the free radical ABTS was performed for the polyphenols in solution (stripped) and loaded in the nPSi-βCD controlled release microparticle. Table 1 provides the results of both the polyphenols in solution. For CA, it was found that there is an inhibition of the ABTS absorbance in all the concentrations tested (50, 150, and 350 μM), and the inhibition% is also directly proportional to the polyphenol concentration used. Thus, significant differences were established when comparing the three concentrations of CA used (*p* < 0.05). The concentration of 50 μM had the lowest ABTS absorbance inhibition% with 10.7 ± 1.7, while 150 μM yielded 24 ± 2.7 and 350 μM 51.8 ± 5.3%, with the latter having the highest inhibition percentage. For the case of Pin, absorbance inhibition was also observed; however, there were no differences between the lowest concentrations, 10 and 20 μM, and the inhibition percentages of 3.4 ± 0.6 and 3.4 ± 0.9, respectively (*p* > 0.05) (Table 1). By contrast, for 65 μM, a slight increase in the inhibition percentage was noted, with values of 4.8 ± 1.3. Therefore, when comparing the antioxidant capacity between the two polyphenols in solution, the caffeic acid clearly had much more scavenger activity than pinocembrin, this results are consistent with the report by Ahn et al. (2009) [58]. These findings suggest that the greater the antioxidant power of the molecule, the greater the antiangiogenic activity.

Other studies link the chemical structure of polyphenols to antioxidant capacity, which establishes that the number of hydroxyls and their position in the chemical structure affords more or less scavenger capacity [59]. In the same way, flavonones have less antioxidant activity because they lack the o-dihydroxy phenyl ring structure, as in the case of Pin. This is not the case with CA, which, being a phenolic acid, possesses various hydroxyl groups in its structure. Finally, some authors propose that the correlation between the antioxidant and antiangiogenic activity may be due to the role of the superoxide and H_2_O_2_ in signal transduction, in response to factors, such as hypoxia, activating multiple pathways that lead, for example, to the migration of endothelial cells [60].

When converting to Trolox equivalents in mg/L, it can be observed that, for CA, the antioxidant capacity changes from medium to high, and for Pin it is very low compared to the gold standard (Trolox) (Table 1). In this sense, CA at 350 μM, the highest concentration used, reached 7.8 ± 0.8 mg/L, indicating more than half of the activity described for Trolox. However, in the greatest concentration used for Pin (65 μM), the values did not exceed 1 mg/L of Trolox.

### 3.7. Kinetics of Antioxidant Capacity of Polyphenols Loaded in the nPSi-βCD Composite Microparticle

**Figure 8:**
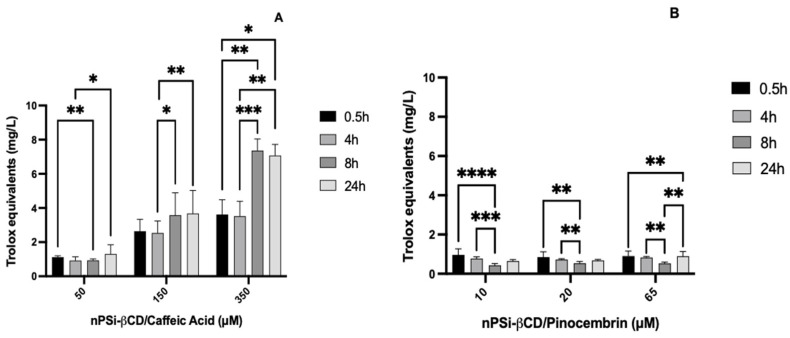
Kinetics of the antioxidant capacity of caffeic acid (**A**) and pinocembrin (**B**) loaded in the nPSi-βCD composite microparticle, expressed in Trolox equivalents (mg/L). The number of asterisks reflects the differences between the concentrations of the microencapsulated polyphenols tested. (* = *p* < 0.05; ** = *p* < 0.01; *** = *p* < 0.001; **** = *p* < 0.0001; *n* = 5).

Given that assays were previously performed to evaluate the controlled release of polyphenols loaded in the nPSi-βCD composite microparticle, the kinetics of the antioxidant capacity of these polyphenols loaded in the microparticle were also evaluated to understand if the antioxidant capacity also occurred gradually. Therefore, the antioxidant capacity of both polyphenols was evaluated now incorporated into the nPSi-βCD controlled release microparticle, and the changes are noted in the ABTS absorbance inhibition % for CA at the different times evaluated (Figure 8).

This indicates the gradual release of the polyphenol over time, reflected in the increase of the antioxidant activity, finding the greatest differences between 4 and 8 h in the concentrations of 150 (17.1 ± 4.6/24.0 ± 8.7) and 350 μM (23.6 ± 5.7 and 48.9 ± 4.5) (*p* < 0.05). However, for the same concentrations, there were no differences in the times 0.5/4 h and 4/24 h. In the case of CA 50 μM, significant differences were noted in the inhibition% obtained between 0.5/4 h (7.7 ± 0.6/6.5 ± 1.4) and 4/24 h (6.5 ± 1.4/9.0 ± 3.5) (*p* < 0.05), although there were no changes between 4/8 h and 8/24 h.

When converting the ABTS absorbance inhibition% to Trolox equivalents in mg/L, the same changes described previously can be found in the scavenger activity (Table 2). In addition, in the kinetics of the antioxidant activity of polyphenols in the nPSi-βCD microparticle, the same behavior was observed as the polyphenols in solution. In other words, nPSi-βCD/CA reaches more than half of the Trolox activity, which results in a greater antioxidant capacity than nPSi-βCD/Pin, which presents a highly reduced activity. Therefore, there are no differences between the polyphenols in solution and those loaded in the nPSi-βCD composite microparticle. In this sense, there are no previous studies that evaluate the kinetics of the antioxidant capacity in polyphenols loaded in microparticles, which is why the results described here constitute the first evidence in this line.

### 3.8. The Effect of the Treatment with Caffeic Acid and Pinocembrin in Solution and Loaded in the nPSi-βCD Composite Microparticle on the Expression of Genes Nrf2, Keap1, Akt, Cat, Glut-P, Hmox-1, Gclc, and Gclm Related to the Antioxidant Pathway

**Figure 9:**
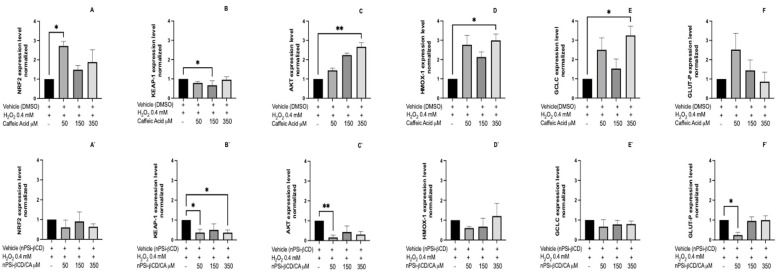
Effect of treatment with caffeic acid on the expression of genes related to the antioxidant pathway. HUVECs were stimulated with 0.4 mM H_2_O_2_ and treated with caffeic acid at concentrations of 50, 150, and 350 μM for 8 h. (**A**–**F**): Treatments with caffeic acid in solution. (**A`**–**F`**): treatments with caffeic acid loaded in the nPSi-βCD composite microparticle. The data were normalized against RPL-30 and GAPDH. The bars represent the mean expression for each group ± standard deviation. The number of asterisks reflects differences between control (vehicle) vs. treatments (* = *p* < 0.05; ** = *p* < 0.01; *n* = 3).

Oxidative stress plays an important role in the pathogeny of many diseases, such as cardiovascular diseases. These evolve with high levels of ROS, which causes damage to the tissue and activation of signaling pathways associated with inflammatory processes. In this context, this study evaluated the expression of genes involved in the antioxidant response. When comparing the changes in the expression of genes involved in the antioxidant response between the control group and the group treated with caffeic acid in solution at different concentrations, we noted differences in Nrf2, Keap1, Akt, Hmox-1, and Gclc (Figure 9A–F). The results show that the treatment with CA 50 μM significantly increases the expression of Nrf2 (*p* < 0.05), whereas, at greater concentrations, there are no differences to the control (Figure 9A). For the case of Keap1 (Figure 9B), a decrease in expression was observed only at a concentration of CA 150 μM (*p* < 0.05). With respect to the expression of Akt (Figure 9C), it can be observed that the treatment with CA increases its expression proportionally to the concentration; however, a significant difference is only noted in the group treated with CA 350 μM (*p* < 0.001).

Finally, Figure 9D,E provide the results for Hmox-1 and Gclc, which show an increased expression level for both genes at concentrations of 50 and 350 μM, with the latter being significant (*p* < 0.05). On the other hand, for Glut-P, no significant changes are observed in the gene expression associated with the treatments (Figure 9F). In the cells exposed to oxidative stress, an increase in the expression of Nrf2 is to be expected, whereas Keap1 would be reduced, which should correspond in these tests to the control condition. Thus, Zhou et al. (2014) [61] proposed that the Keap-1-dependent Nrf2 system is activated in cells under conditions of oxidative stress, causing Keap-1 to degrade and Nrf2 to translocate to the nucleus and causing the transcriptional activation of genes associated with antioxidant and detoxifying enzymes. In addition, these genes promote cytoprotective mechanisms, such as cell proliferation and angiogenesis. Therefore, by understanding the antiangiogenic effect exhibited by CA and Pin in this and other studies, the treatment with these polyphenols should manifest changes in the expression of these genes. However, the results observed do not account for this effect. This finding could be related to the loss of antioxidant activity of the polyphenols in solution, which emphasizes the potential utility of loading them in microparticles.

The effect of the microencapsulation of CA on changes in the gene expression of HUVECs treated with the nPSi-βCD + polyphenol microparticle was evaluated. Among the results for nPSi-βCD/CA (Figure 9A`,F`), a reduction in the expression of Keap-1 is observed in the group treated with nPSi-βCD/CA 50 and 350 μM (*p* < 0.05) (Figure 9B`), while a decrease is also noted for nPSi-βCD/CA 150 μM, but it is not significant. For the case of Akt and Glut-P (Figure 9C`,F`), there is a reduction in the expression level in both genes, mainly in the group treated with nPSi-βCD/CA 50 μM (*p* < 0.05), in contrast to those treated with nPSi-βCD/CA 150 and 350 μM, where an increase in expression is observed, which is not significant. Finally, no differences are found in the gene expression of Nrf2, Hmox-1, Gclc (Figure 9A`,D`,E`).

### 3.9. The Effect of the Treatment with Caffeic Acid and Pinocembrin in Soluion and Loaded in the nPSi-βCD Composite Microparticle on the Expression of Genes Nrf2, Keap1, Akt, Cat, Glut-P, Hmox1, Gclc, and Gclm Related to the Antioxidant Pathway

**Figure 10:**
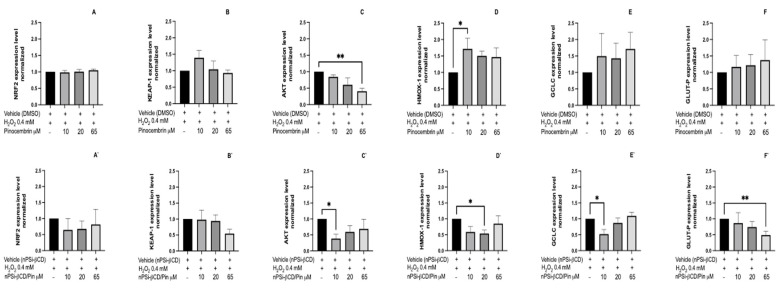
Effect of treatment with pinocembrin on the expression of genes related to the antioxidant pathway. HUVECs were stimulated with 0.4 mM H_2_O_2_ and treated with pinocembrin at concentrations of 10, 20, and 65 μM for 8 h. (**A**–**F**): Treatments with pinocembrin in solution. (**A`**–**F`**): treatments with pinocembrin loaded in the nPSi-βCD composite microparticle. The data were normalized against RPL-30 and GAPDH. The bars represent the mean expression for each group ± standard deviation. The number of asterisks reflects differences between vehicle vs. treatments (* = *p* < 0.05; ** = *p* < 0.01; *n* = 3).

In contrast to what was found for CA, the treatment with Pin in solution only brought changes in the expression of Akt and Hmox-1 (Figure 10). In the first case, there is a reduction in the expression level proportional to the Pin dose used; however, this is significant at 65 μM (*p* < 0.01) (Figure 10C). By contrast, for Hmox-1, the treatment with Pin 10 μM leads to a significant increase in expression (*p* < 0.05) (Figure 10D), whereas for 20 and 65 μM, no changes are observed compared to the control (vehicle). The same can be observed for Nrf2, Keap-1, Gclc, and Glut-P, where no changes are found in the expression levels (Figure 10A,B,E,F).

On the other hand, for the nPSi-βCD/Pin composite microparticle, the results show no significant changes in the gene expression of Nrf2, Keap-1, and Gclc (Figure 10A`,B`,E`). For Akt, there is a significant reduction in gene expression, only in the group treated with nPSi-βCD/Pin 10 μM (Figure 10C`) (*p* < 0.05). The same results are found for Gclc, as shown in Figure 9E` (*p* < 0.05); however, for nPSi-βCD/Pin 20 and 65 μM, the opposite effect can be observed, since the expression tends to continue increasing without ever being significant. With respect to Glut-P, the treatment with nPSi-βCD/Pin causes a significant decrease in expression in the group treated with nPSi-βCD/Pin 65 μM (*p* < 0.01); there are no differences for the other concentrations (Figure 10F`). Finally, a reduction in the expression of the Hmox-1 gene is also noted, only for the group treated with nPSi-βCD/Pin 20 μM (Figure 10D`). Thus, these results indicate no changes in the expression of genes related to the antioxidant pathway in HUVECs treated with polyphenols incorporated into the nPSi-βCD composite microparticle. The results of this study show that the antiangiogenic activity of caffeic acid, evaluated by Matrigel and CAM, is greater than that observed in pinocembrin. In this context, the greatest changes in the gene expression were observed in cells treated with nPSi-βCD/CA 50 μM. By understanding the biological antiangiogenic activity of CA, it is expected that the treatment will generate a decrease in Keap-1, Akt, and Glut-P.

## 4. Conclusions

The polyphenols caffeic acid and pinocembrin present antioxidant and antiangiogenic activity in solution and microencapsulated. The biological effect is greater for caffeic acid than for pinocembrin, especially when incorporated into the nPSi-βCD composite microparticle. Finally, this work demonstrated that the use of the nPSi-βCD composite microparticle as a controlled release system of the two polyphenols is a good strategy for transporting biopolymers, as it improves their biological activity. Therefore, the use of this controlled drug release system could be used for future antiangiogenic and antioxidant therapies for chronic non-communicable diseases, such as cardiovascular diseases.

## Figures and Tables

**Table 1:** Antioxidant capacity of caffeic acid and pinocembrin in solution.

Polyphenol in Solution	Concentration (μM)	% ABTS Absorbance Inhibition	Trolox Equivalents (mg/L)
**Caffeic acid**	50	10.7 ± 1.7 _a_	1.6 ± 0.3
	150	24.0 ± 2.7 _b_	3.6 ± 0.4
	350	51.8 ± 5.3 _c_	7.8 ± 0.8
**Pinocembrin**	10	3.4 ± 0.6 _a_	0.5 ± 0.1
	20	3.4 ± 0.9 _a_	0.5 ± 0.1
	65	4.8 ± 1.3 _b_	0.7 ± 0.2

Data were expressed as the percentage inhibition of absorbance at 734 nm wavelength of the free radical ABTS^•^ and Trolox equivalents (mg/L) (mean ± standard deviation). Lowercase letters next to the mean and standard error values indicate the results of the statistical analysis (*p* < 0.05, *n* = 9).

**Table 2:** Kinetics of the antioxidant capacity of caffeic acid and pinocembrin loaded in the nPSi-βCD composite microparticle.

Microencapsulated Polyphenol	% ABTS Absorbance Inhibition				Trolox Equivalents (mg/L)			
	0.5 h	4 h	8 h	24 h	0.5 h	4 h	8 h	24 h
CA 50	7.7 ± 0.6	6.5 ± 1.4	6.5 ± 0.6	9.0 ± 3.5	1.1 ± 0.1	0.9 ± 0.2	0.9 ± 0.1	1.3 ± 0.5
CA 150	17.8 ± 4.6	17.1 ± 4.6	24.0 ± 8.7	24.7 ± 8.9	2.6 ± 0.7	2.5 ± 0.7	3.6 ± 1.3	3.7 ± 1.4
CA 350	24.3 ± 5.7	23.6 ± 5.7	48.9 ± 4.5	47.0 ± 4.3	3.6 ± 0.9	3.5 ± 0.9	7.4 ± 0.7	7.1 ± 0.7
Pin 10	6.7 ± 2.0	5.5 ± 0.6	3.2 ± 0.6	4.7 ± 0.5	0.1 ± 0.3	0.8 ± 0.1	0.4 ± 0.1	0.7 ± 0.1
Pin 20	6.0 ± 1.8	5.1 ± 0.3	3.9 ± 0.6	4.9 ± 0.3	0.9 ± 0.3	0.7 ± 0.0	0.5 ± 0.1	0.7 ± 0.1
Pin 65	6.4 ± 1.7	5.8 ± 0.4	3.9 ± 0.4	6.3 ± 1.6	0.9 ± 0.3	0.8 ± 0.1	0.5 ± 0.1	0.9 ± 0.2

The data were expressed as a percentage of inhibition of absorbance at 734 nm wavelength of the free radical ABTS^•^ (mean ± standard deviation) (*p* < 0.05; *n* = 9).

## Data Availability

Not applicable.
